# Recent Bioinformatics Advances in the Analysis of High Throughput Flow Cytometry Data

**DOI:** 10.1155/2009/461763

**Published:** 2010-02-09

**Authors:** Raphael Gottardo, Ryan R. Brinkman, George Luta, Matt P. Wand

**Affiliations:** ^1^Computational Biology Unit, Clinical Research Institute Montréal (IRCM), 110 Avenue des Pins Ouest, Montréal, Québec, Canada H2W 1R7; ^2^BC Cancer Agency, 675 West 10th Avenue Vancouver, Canada BC V5Z 1L3; ^3^Department of Biostatistics, Bioinformatics, and Biomathematics, Georgetown University, Medical Center, 4000 Reservoir Rd., NW Building D, Suite 180 Washington, DC 20057-1484, USA; ^4^School of Mathematics and Applied Statistics, University of Wollongong, Northfields Avenue, Wollongong 2522, Australia

For more than 30 years, the fluorescence-based technique of flow cytometry (FCM) has been widely used by clinicians, immunologists, and cancer biologists to distinguish different cell types in mixed cell subpopulations, based on the expression of cellular markers. In both health research and treatment, this analytical method is used for a variety of tasks, in particular the diagnosis and monitoring of cancer. This technology is also used for cross-matching organs for transplantation, and for research involving stem cells, vaccine development, apoptosis and phagocytosis.

In the last decade, advances in FCM instrumentation and reagent technologies have enabled simultaneous single cell measurement of surface and intracellular markers, including cellular-activation markers, intracellular cytokines, immunological signaling, and cytoplasmic and nuclear cell cycle and transcription factors, thus positioning FCM to play an even bigger role in health care and medical research. 

However, the rapid expansion of FCM applications has outpaced the development of tools for storage, analysis, and data representation. For example, a typical FCM experiment may involve measurement of up to 20 different characteristics per cell, for hundreds of thousands of cells per sample. The increase in the amount of data generated by FCM techniques poses unique informatics and statistical challenges. 

It is widely recognized that one basic challenge for FCM is to simplify the extraction of data and statistical information. To date, very few bioinformatic and statistical tools exist to manage, analyze, present, and disseminate FCM data. Current FCM data analysis methods involve the use of multiple applications, the output of which is often fragmented. There is a widespread demand for the development of integrated data analysis tools to organize, analyze, and exchange FCM data. Such development is lagging far behind the ability to collect and process samples via FCM, much to the detriment of health research.

This special issue aims to summarize the current state of bioinformatics research in FCM, to present the most recent developments in analytical tools and to open-up the field to new researchers to bring additional ideas and solutions to current bottlenecks. The issue includes several important contributions, which cover a wide range of approaches and techniques for FCM. These contributions are summarized as follows.

Bashashati and Brinkman review state-of-the-art FCM data analysis approaches that can be used in a typical analysis pipeline going from quality assessment to sample classification. Not only does their paper review current techniques and approaches but it also points out potential pitfalls of these approaches and discusses strategies to overcome these.

Much like with gene expression data, technical variation such as changes in the instrumentation channel voltages or changes in the specificity of the manufacturer of the antibodies can result in systematic biases. These biases need to be removed or at least minimized in order to allow proper data analysis and sample comparisons. Cichocki et al. present a novel normalization method to correct for time biases in large-scale flow cytometric analysis. They investigate two types of normalizing beads: broad spectrum and spectrum matched and propose two alternative normalization procedures that are usable in the absence of normalizing beads.

Once data have been properly normalized, a component of FCM analysis involves identifying immunophenotypically distinct sub-populations of cells within each patient; this is referred to as “gating” in the FCM community. Although gating has traditionally been done visually, automated approaches based on statistical modeling of the data are starting to emerge. Walther et al. present such an approach based on a nonparametric statistical model that aims to form cell subpopulations that can be delineated by the contours of high-density regions much like in manual gating. Because their approach is non-parametric it can reproduce non-convex subpopulations that are known to occur in FCM samples, but which cannot be produced with current parametric model-based approaches. Much like Walther et al., Finak et al. present a framework for the identification of cell subpopulations in FCM data based on merging mixture components using the *flowClust* methodology. In this new approach, several parametric clusters can represent a single sub-population, and the approach can thus accommodate complicated FCM data distributions (e.g., non-convex sub-populations).

Even though automated gating methods are becoming increasing popular, the majority of FCM experiments are still being analyzed visually, usually by serial inspection of one or two dimensions at a time. In order to improve and validate automated gating, it is important to compare automated gates to manual gates obtained by an expert. Gosink et al. introduce a Bioconductor package called flowFlowJo that can import gates defined by the commercial package FlowJo and work with them in a manner consistent with the other flow packages in Bioconductor. This work facilitates examination of gating robustness, allows one to combine manual and automated gating, and can be used to perform exploratory data analysis on manual gates.

Another major goal in clinical applications is the identification of biological changes (e.g., proportion of cells within a subpopulation) that correlate with a disease in order to predict the status (e.g., healthy/diseased) of a patient. Rogers and Holyst present flowFP, a Bioconductor package for fingerprinting flow cytometric data. flowFP provides tools to transform raw FCM data into a form suitable for direct input into conventional statistical analysis and empirical modeling software tools (e.g., supervised classification). Among other things flowFP is based on a multivariate binning approach and thus can bypass the gating stage, which can be an advantage for complex flow data.

In a similar clinical context, Eliot et al. investigate the use of tree-based methods for discovering associations between flow cytometry data and clinical endpoints. In particular, they compare a number of tree-based methods for their capability to select immunological predictors of CD4 reconstitution in HIV-infected subjects initiating anti-retroviral treatment. The authors show that tree-based methods can be successfully applied to flow cytometry data to better inform and discover associations that may not emerge in the context of a standard univariate analysis.

Even though Bioconductor is a great platform for FCM allowing computational statisticians and bioinformaticians to leverage the power of R and other contributed packages, it can remain difficult to be used by biologists and clinicians. Lee et al. have developed an open source, extensible graphical user interface (GUI) iFlow, which sits on top of the Bioconductor backbone, enabling basic analyses by means of convenient graphical menus and wizards. iFlow is easily extensible in order to quickly integrate novel methodological developments.

Finally, Strain et al. introduce plateCore, a new package that extends the functionality of core FCM Bioconductor packages to enable automated negative control-based gating and facilitate the processing and analysis of plate-based data sets from high-throughput FCM screening experiments.

